# Detection of Bacterial Metabolic Volatile Indole Using a Graphene-Based Field-Effect Transistor Biosensor

**DOI:** 10.3390/nano11051155

**Published:** 2021-04-28

**Authors:** Zihong Lin, Guangfu Wu, Ling Zhao, King Wai Chiu Lai

**Affiliations:** 1 Department of Biomedical Engineering, Centre for Robotics and Automation, City University of Hong Kong, Kowloon Tong, Hong Kong 999077, China; zihonglin2-c@my.cityu.edu.hk (Z.L.); lingzhao3-c@my.cityu.edu.hk (L.Z.); 2 Department of Biomedical Engineering, University of Connecticuit, Storrs, CT 06269, USA; guangfu.wu@uconn.edu

**Keywords:** indole, graphene, field-effect transistor, indole detector, bacterial metabolic indole

## Abstract

The existence of bacteria is a great threat to food safety. Volatile compounds secreted by bacteria during their metabolic process can be dissected to evaluate bacterial contamination. Indole, as a major volatile molecule released by *Escherichia coli* (*E. coli*), was chosen to examine the presence of *E. coli* in this research. In this work, a graphene field-effect transistor (G-FET) was employed to detect the volatile molecule-indole based on a π-π stacking interaction between the indole and the graphene. The exposure of G-FET devices to the indole provokes a change in electrical signal, which is ascribed to the adsorption of the indole molecule onto the graphene surface via π-π stacking. The adsorption of the indole causes a charge rearrangement of the graphene-indole complex, which leads to changes in the electrical signal of G-FET biosensors with a different indole concentration. Currently, the indole biosensor can detect indole from 10 ppb to 250 ppb and reach a limit of detection of 10 ppb for indole solution detection. We believe that our detection strategy for detecting bacterial metabolic gas molecules will pave a way to developing an effective platform for bacteria detection in food safety monitoring.

## 1. Introduction

**Figure 1:**
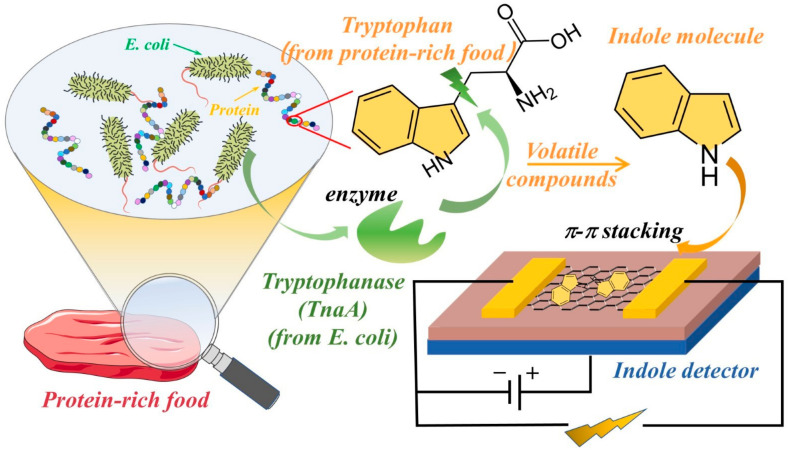
The schematic of metabolic volatile indole detector using G-FET.

Pathogenic bacteria significantly impact human health and the environment due to their ability to cause food-borne illness [1,2,3]. More than 2 million people die from unsafe foods every year, according to the 2015 WHO report [4]. Various methods have been developed to directly or indirectly detect foodborne pathogenic bacteria [5,6,7,8]. The direct methods, such as viable cell enumeration [9], the selective isolation of bacteria on commercial media [10], and immunoassays [11], can directly detect bacteria, which is often tenuous with molecular-based technologies. Using these conventional methods is time-consuming for sample pretreatment and the proliferation of pathogenic bacteria [12]. Additionally, even though these agents and the equipment for detecting bacteria are commercially available, significant fluctuations in the detection results often happen while using these methods. It is of great importance to develop a novel method to detect bacteria rapidly and simply. Therefore, some indirect methods have been developed [13,14] which translate the direct detection of bacteria into the detection of their metabolites, such as volatile organic compounds (VOCs) [15,16]. Generally, spoiled food has an unpleasant odor, which can be used for the identification of food quality [17,18]. Specifically, various bacteria can produce different VOCs. For example, yeast can transform polysaccharide into volatile alcohol, which indicates fruit spoilage [19]. *Escherichia coli* (*E. coli*) can transform amino acids, such as tryptophan, into indole, which is extensively present in milk, meats, and seafood, as shown in Figure 1.

Researchers have tried to improve the performance of the detection of bacteria by proposing various strategies based on VOC detection [20,21]. For example, an approach was developed to detect *Listeria monocytogenes* contamination of milk samples by analyzing enzyme-generated VOCs, the metabolic volatile 2-nitrophenol, and the 3-fluoroaniline of *Listeria monocytogenes* [20]. However, although they are relatively fast for the identification of bacterial contamination in food compared to direct detection approaches, these indirect methods still rely on expensive equipment such as gas chromatography/mass spectrometry (GC/MS) [22] and fluorescence spectroscopy [23], which require professional operation.

Therefore, to avoid the utilization of large-scale equipment and shorten the detection time of VOCs, it is necessary to develop portable electronic devices based on the olfactory sense to separate the volatile compound from bacteria. Graphene has a lot of advantages, including its large surface to volume ratio [24], high carrier mobility [25], and low noise characteristics [26]. It also has a large π bond and can interact with indole through a π-π interaction [27]. In addition, graphene has unique optical, mechanical, and electrical properties [28], and is commonly used as the conductive channel between the source and drain electrodes [29] in graphene-based field-effect transistor (G-FET) sensors [30,31]. Since the absorption of molecules on graphene surfaced can increase the charge redistribution and the physical doping of graphene, graphene-based sensors are very sensitive in analyte detection [32]. To enhance the detection performance of G-FET, various gate structures were developed to regulate its conducting behavior, such as back gate G-FET [29], top gate G-FET [33,34], and solution gate G-FET [35].

In this study, we developed an indole detector using a G-FET biosensor to detect the VOCs and metabolic indole molecules produced by *E.coli*. using a portable electronic device based on olfactory, graphene-based field-effect transistors (G-FET) that can generate electrical signals during the detection process. The exposure of G-FET devices to the indole induces changes in the electrical signal, which is ascribed to the adsorption of the indole molecule onto the graphene surface via π-π stacking. The adsorption of the indole causes a charge rearrangement of the graphene-indole complex, which leads to changes in the electrical signal of the G-FET biosensors with the different indole concentrations.

## 2. Materials and Methods

### 2.1. Indole Preparation

**Figure 2:**
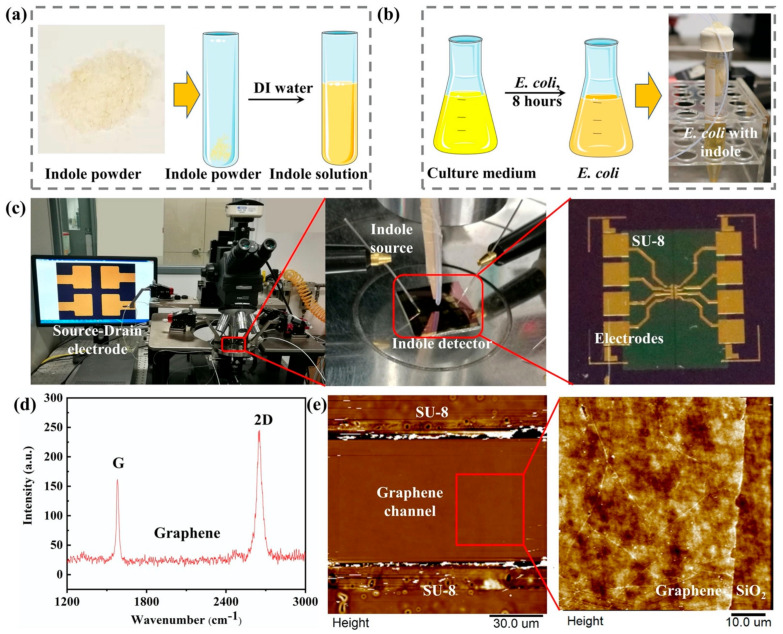
(**a**) The preparation of the as-prepared indole solution. (**b**) The preparation of metabolic indole from *E. coli*. (**c**) The optical image of a G-FET indole detector and the experimental setup of the indole detection on a probe station. (**d**) Raman spectroscopy of graphene. (**e**) AFM image of the graphene channel.

Before the indole detection experiment, the indole sample was prepared according to the nature of the experiments. The indole samples mainly came from two sources: one came from the indole power (as-prepared indole) and the other from *E. coli* (metabolic indole). For the as-prepared indole solution detection, 10 mg of indole powder was directly diluted into 100 mL of DI water to prepare the as-prepared indole solution with a concentration of 100 ppm, as shown in Figure 2a. Then, different concentrations of the indole solution ranging from 10 ppb to 1 ppm were obtained by diluting the high-concentration indole solution. For the as-prepared indole gas detection, 1 g of indole powder was added to an airtight conical tube connected to a silicone tube with a diameter of 1 mm to obtain indole gas.

In the bacterial metabolic experiment, indole was obtained from the *E. coli* metabolic process, as shown in Figure 2b. A quantity of 0.8 g of broth and 0.00015 g of streptomycin was dissolved in 60 mL of deionized water to form the *E. coli* bacterium medium solution with a concentration of 1.3% (*w/v*) in a conical flask. Then, the conical flask was transferred into the autoclave to sterilize and exclude the contamination of bacteria present in the air. After sterilizing, the *E. coli* K12 was taken out from the −70 °C freezer and transferred into a 37 °C water bath. Then, the *E. coli* was inoculated into a culture medium. After culturing for 8 h in a shaking incubator at a temperature of 37 °C and shaking at a speed of 110 rounds per minute, the growth of the *E. coli* reached the stationary phase in the culture media. A total of 0.204 g of tryptophan was added to the *E. coli* culture media. Then, the conical flask was transferred into the shaking incubator and cultured to metabolize the tryptophan into indole molecules.

### 2.2. Design of Indole Detector

**Figure 3:**
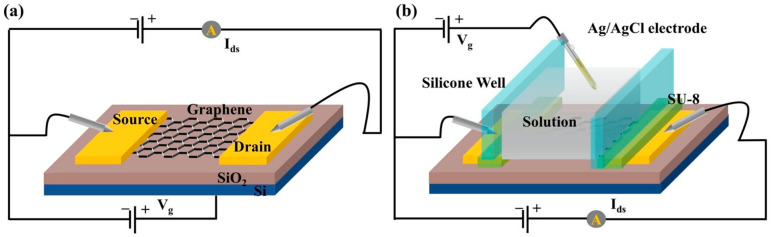
(**a**) The back-gate FET for gas-phase indole detection. (**b**) The solution-gate FET for liquid-phase indole detection.

We designed graphene-FET as the indole detector, which mainly includes the graphene channel connected to gold source-drain electrodes. Two kinds of G-FET sensors were designed with different gate structures (back gate and solution gate) for the air-phase indole and the liquid-phase indole, as shown in Figure 3.

**Figure 4:**
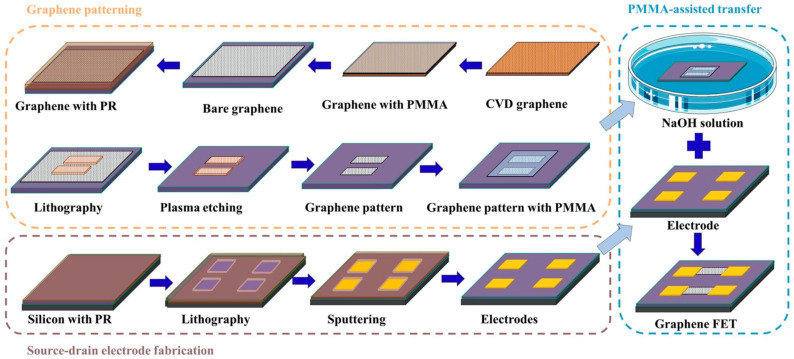
The fabrication process of the indole detector.

The device fabrication mainly included two parts: the fabrication of the source-drain electrodes and graphene patterning, as shown in Figure 4. Firstly, cleaned silicon wafer (*p*-type, 100 crystallographic orientations, 0.001–0.005 Ω cm resistivity) with a 300 nm SiO_2_ (Zhejiang Lijing Materials Technology Co., Zhejiang, China) was used as a substrate for the source and drain electrode fabrication. The metal electrodes were made by a conventional lithography process that started with the spin coating of photoresist (AZ5214) on the wafer, followed by baking at 90 °C for 3 min on a hot plate. Afterwards, it was exposed to a mask aligner at a wavelength of 365 nm for 7 s. The patterns were then developed by using a resist developer/DI water solution with a volume ratio of 1:3. Then, 15 nm Cr and 90 nm Au were deposited on the wafer by RF sputtering. The substrate with source-drain electrodes was ready after immersing the wafer in acetone for 1 h to remove the remaining photoresist. Secondly, a monolayer chemical vapor deposition (CVD) grown graphene with a lateral size of 100 μm was adopted as the conductive channel. The CVD graphene on copper foil was a commercial sample purchased from Xiamen G-CVD Technology Co., Ltd., Fujian, China. To achieve the repeatability of the G-FET sensor, a well-shaped uniform graphene pattern was obtained by photolithography with a size of 100 × 100 μm. PMMA-assisted graphene transfer was adopted for graphene patterning [36]. This was started by spin-coated graphene on copper foil with a layer of PMMA with a concentration of 20 mg/mL. Next, the graphene/PMMA film was immersed in an Iron (III) Chloride Hexahydrate solution (FeCl_3_·6H_2_O, 1 M) to etch the underneath copper foil, followed by washing the film sequentially with a 5% HCl solution and deionized water. Then, it was carefully transferred on a silicon wafer followed by a drying procedure at a temperature of 80 °C. This substrate was further immersed in acetone for 24 h to remove the PMMA film, and bare graphene was obtained. The graphene film obtained on the silicon substrate was spin-coated with a layer of photoresist and cured at a temperature of 90 °C. After solidification, the photoresist-covered graphene was exposed to a UV light for 9 s using a photomask, followed by a developing process. Then, the chip was transferred to oxygen plasma for 20 min. The uncovered parts of graphene were oxidized and removed. Lastly, the substrate was immersed in acetone to remove the photoresist, and a graphene pattern with a certain size was obtained. Finally, after the preparation of the source-drain electrode and the graphene pattern, the graphene pattern was transferred to the metal electrode by the Figure 2c. Raman spectroscopy and atomic force microscopy (AFM) were used to characterize the monolayer graphene on the SiO_2_/Si wafer, as shown in Figure 2d,e, respectively. According to the research, the G and 2D peaks of the monolayer graphene Raman spectra G are at around 1582 cm^−1^ and the 2D peak at around 2629.7 cm^−1^ can be fitted with a single Lorentzian line shape [37], which demonstrates that the graphene is unilaminar. Moreover, the morphology of the graphene channel was also investigated by AFM. As can be observed from the AFM image (Figure 2e), the graphene film was continuous and uniform [31].

### 2.3. Electrical Measurements

The electrical characterizations of G-FETs were conducted using a semiconductor device analyzer (Semiconductor Analyzer, B1500, Keysight Co. Ltd., Santa Rosa, CA, USA) coupled with a probe station. The source and drain electrodes were connected to the semiconductor analyzer, which could provide a voltage to our device by two probes on the probe station, as shown in Figure 2c. The probes could move from x, y, and z and in three directions by adjusting the manipulators with an accuracy at the micrometer level to achieve a proper position to make good contact with the two gold electrodes. The source electrode ground as a reference potential and a voltage (V_ds_) were applied to the source and drain electrodes with a value of 100 mV. The gate voltages (V_g_) of the solution and gas detection were 0.1 V and 25 V, respectively.

## 3. Results and Discussion

### 3.1. Indole Solution Detection

**Figure 5:**
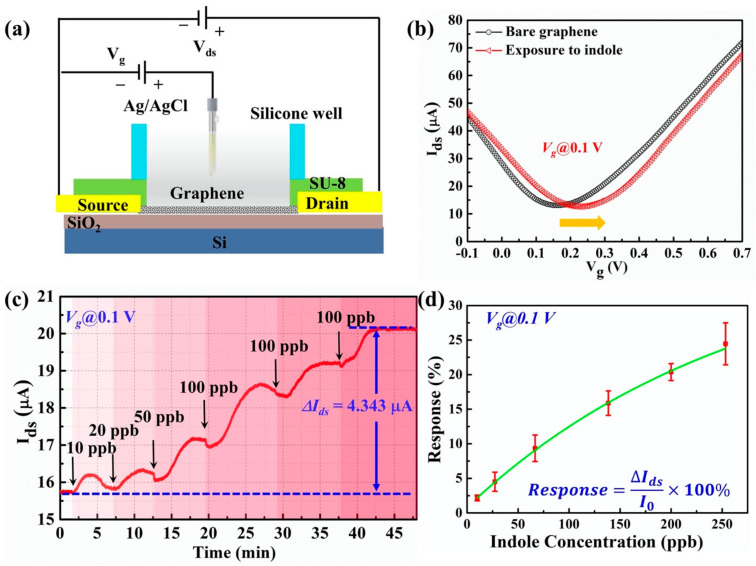
(**a**) The experimental setup of liquid-phase indole detection. (**b**) The transfer characteristics of graphene channel before and after the exposure of indole. (**c**) The real-time detection of indole solution using the G-FET biosensor. (**d**) The correlation between the electrical responses and various concentrations of the indole solution.

During the experiment for the detection of liquid-phase indole using G-FET, the source and drain electrodes of the G-FET were first insulated by SU-8 to prevent possible leakage current from the solution during the detection process. After that, silicone rubber was used to form a recording chamber on the device’s surface, in which graphene was introduced to detect indole molecules, as shown in Figure 5a.

Firstly, the characteristic transfer curve of the as-prepared G-FET device was characterized before and after the interaction of liquid-phase indole molecules. As shown in Figure 5b, the black and red lines represent the transfer curves of graphene before and after exposure to indole. A right shift of the transfer curve was observed, which indicated that indole could interact with the graphene channel and impose a *p*-doping effect of graphene. The shift in the Dirac point, resulting from a change in the electric charge on the graphene surface, confirmed the successive surface attachment of indole molecules. The results showed that the real-time *I_ds_* could reflect the state of the molecular adsorption on the graphene surface. Secondly, from the transfer curve the carrier mobility of hole carriers reached the maximum value at a gate voltage equal to 0.1 V. Therefore, a real-time detection experiment will be conducted with the gate voltage of 0.1 V. Various concentrations (10 ppb, 20 ppb, 50 ppb, and 100 ppb) of the indole solution were added to our G-FET biosensor. In each drop, the volume of the solution was 10 µL, so the final concentration of the indole solution on the sensor was estimated to be 253 ppb. The real-time detection result is shown in Figure 5c. After each drop of indole solution, the signal of the sensor increased because of the attachment of the indole molecules to the surface of the graphene. It was also found that the change in current (*∆I_ds_*) was related to the concentration of the indole solution. When the concentration was at 100 ppb, the *∆I_ds_* turned to the highest value. However, further adding of the indole solution did not lead to a higher signal change because the device was saturated at the end of each experiment. As the attachment of the indole molecules on the graphene surface was a complex process, some indole molecules also disassociated from the graphene surface, resulting in an equilibrium of association and disassociation. To gain a better understanding of the relationship between the electrical response and the concentration of the indole sample, we analyzed the electrical responses by repeating the experiment with multiple sensors (*n* = 5). The electrical response was defined as the percentage ratio of *∆I_ds_* over the initial source-drain current, as shown in Figure 5c. For different concentrations of indole, the response changes are summarized in Figure 5d. The electrical response and the indole concentration have a good linear relationship, especially at low concentrations. When the concentration of indole was low, all indole molecules could easily attach to the graphene surface because sufficient vacant binding sites were provided. However, when the concentration of indole became very high, the indole molecules adsorbed on the graphene surface tended to saturate due to the finite surface area of the graphene, which was why the response–concentration curve no longer obeyed a linear relationship. The results demonstrate that our G-FET sensors can be used for quantitative liquid-phase indole detection.

### 3.2. Indole Gas Detection

**Figure 6:**
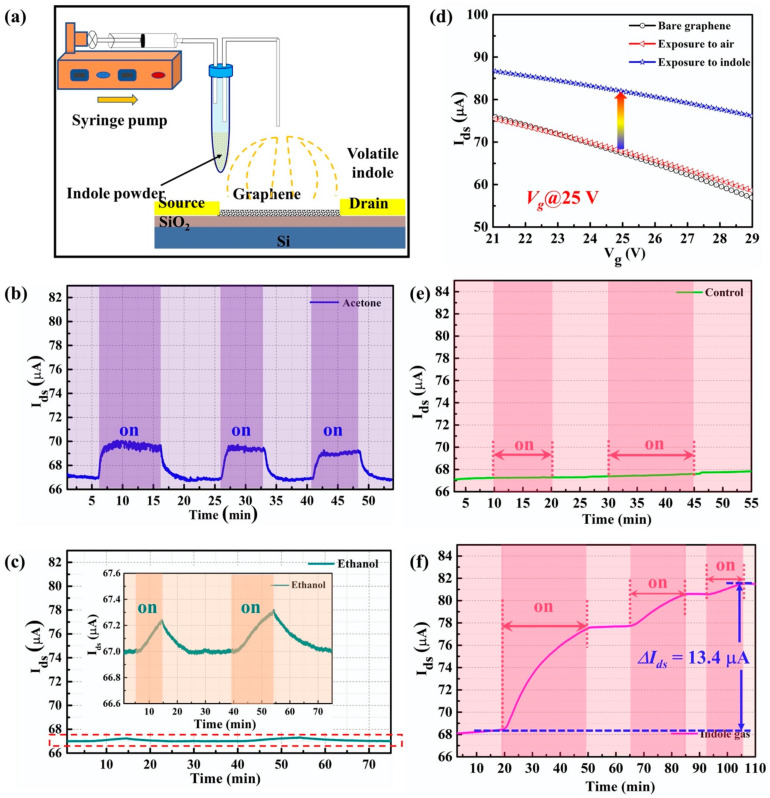
(**a**) The experimental setup of gas-phase indole detection. (**b**) The real-time response of acetone using the G-FET biosensor. (**c**) The real-time response of ethanol using the G-FET biosensor. (**d**) The transfer characteristics of graphene channel before and after the exposure of the gas-phase indole. (**e**) The control experiment using air gas. (**f**) The real-time detection of indole gas molecules using the G-FET biosensor.

To detect the gas-phase indole, the experimental setup is demonstrated in Figure 6a. The back gate was adopted, and the gate voltage was applied to the backside of the silicon directly. The indole solid was put into a plastic test-tube which was connected to two tubules. The short tubule was connected to a syringe which was driven by a syringe pump. The long tubule was fixed on top of our G-FET biosensor. During the detection experiment, the syringe pump was operated at a rate of 200 μL/min, in order for the volatile indole to reach the surface of the G-FET biosensor. The experiments were conducted using a temperature of 20 °C. Since *E. coli* can produce various metabolites which can interfere with indole detection, such as different types of alcohols and esters of alcohols, we investigated the interference experiments of alcohols and ketones to observe the influence of hydroxy and carbonyl groups. Both alcohol and ketones can provoke small changes in the electrical signal, but the signal change (*ΔI_ds_*) disappeared upon the ceasing of exposure of our sensor to alcohols and ketones (Figure 6b,c). However, even after the pump was stopped, the signal change (*ΔI_ds_*) induced by the indole was constant.

As the ambient condition of indole gas detection is under an atmosphere, a control experiment was conducted using air gas, as shown in Figure 6e. The real-time result demonstrates that before and after the air interacts with the graphene surface, there is no obvious change in the electrical properties of the graphene. Moreover, the characterization of the transfer curve followed, as shown in Figure 6d. The dark line barely shows changes compared to the transfer curve (red line) of the bare graphene, which also indicated that the electrical signal was not influenced by the airflow. The real-time detection of indole gas was conducted at the gate voltage of 25 V. As shown in Figure 6f, when the syringe pump was turned on, the source-drain current (*I_ds_*) visibly increased. However, the *I_ds_* tended to keep constant when the pump was stopped. The real-time response tended to be flat around 81.5 µA and led to a signal change of 13.4 µA, which showed a response of 19.7% for the gas-phase indole detection. Indole is an aromatic heterocyclic organic compound consisting of a six-membered benzene ring fused to a five-membered pyrrole ring. It can be absorbed on a graphene surface due to the π-π interactions attributed to its molecule structure [38], and leads to a signal change in G-FET. This result demonstrated that our G-FET sensor can be utilized for indole gas detection. Moreover, the transfer curve after the indole detection was described. The transfer curve obviously shifted to the right, as shown in Figure 6d (blue line), which indicates that the indole molecule has a *p*-doping effect on the graphene channel. This result was consistent with the liquid-phase indole detection and indicated that the G-FET sensor can effectively detect the indole molecules.

### 3.3. Bacterial Metabolic Indole Detection

As indole is a metabolic volatile organic compound (VOC) of *E. coli*, it is possible to detect the presence of *E. coli* by detecting the metabolic volatile indole. The *E. coli* metabolic indole can be present both in an *E. coli* culture medium and its surrounding air. In this experiment, we investigated the detection of the liquid-phase and air-phase metabolic indoles.

**Figure 7:**
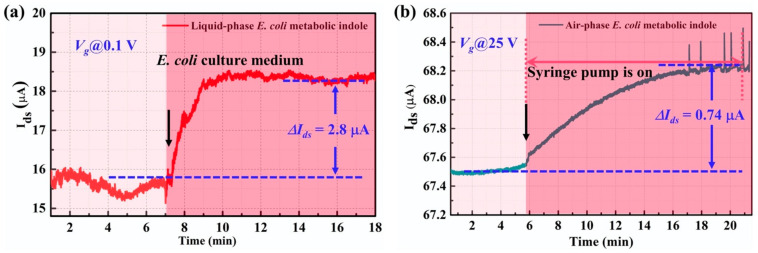
The real-time detection of metabolic volatile indole of *E. coli* in (**a**) liquid phase and (**b**) gas phase.

For the detection of indole in an *E. coli* culture medium, the culture medium of *E. coli* that contains metabolic indole was directly added to our G-FET sensor. The real-time electrical response is shown in Figure 7a. The *ΔI_ds_* caused by the *E. coli* metabolic indole was about 2.8 μA, which demonstrates a response of 17.5%. This result indicated that the G-FET biosensor has the potential to be utilized for bacteria detection by detecting bacteria in the metabolic culture medium. Moreover, the *E. coli* metabolic volatile indole gas was also detected in our experiment by putting the *E. coli* culture medium in a conical tube using our indole gas detector. A filter paper with desiccant was located in the middle of the conical tube to minimize the influence of water molecules. The real-time electrical response of the sensor is shown in Figure 7b. The *E. coli* metabolic volatile compounds caused a current change of 0.74 µA, which showed a response of 1.09%. This result demonstrates that our back-gate G-FET biosensor can be utilized for volatile indole gas sensing. By combining the two sensing modes, the indole in the bacteria culture medium and the volatile indole gas, our G-FET biosensor may have the capacity to be used in the field of food safety.

Various methods have been reported for the detection of indole, as shown in Table 1, including optical, electrochemical, and color-based methods. The optical sensor reported by Sabine Crunaire et al. can detect a wide range of indole from 120 ppb to 30 ppm [39]. However, its low concentration indole detection capacity is limited. Kovac’s or Ehrlich’s reagent is also a commonly used reagent for qualitative indole detection which uses a solution that changes colors from yellow to cherry red [40]. Nevertheless, it can only give a positive or negative result without any quantification of indole concentration. Beyond these methods, an electrochemical sensor was also adopted to detect indole in a liquid-phase with a detection range of 0.5–120 ppb. Compared to the reported methods, our G-FET biosensor can not only detect indole in a solution phase with a detection range of 10–250 ppb, but it can also detect indole in a gas phase, which demonstrates that our G-FET design strategy for indole detection is likely to develop an effective platform for bacteria detection in food safety monitoring.

## 4. Conclusions

We presented a G-FET biosensor that enables the label-free detection of the indole molecule, which is a metabolic product of *E. coli*. To achieve the label-free detection of low-charged small indole molecules, we adopted graphene as the sensing material to provide a high sensitivity in electrical measurements. The sensing mechanism was based on the π-π stacking interaction between indole and graphene which can affect the electrical signal of the graphene device. The attachment of the indole on the graphene surface generated a change in the source-drain current. Consequently, the existence of an indole molecule led to a change in the electrical conductance of the graphene. The detection limit of the indole in our G-FET sensor is as low as to 10 ppb. In conclusion, our G-FET biosensor can potentially be used in the quantitative and label-free detection of low-charged small-molecules.

## Figures and Tables

**Table 1:** The performance of various reported indole sensing methods.

Type of Sensors	Signal	Detection Range	LOD
FET sensor (this study)	Current (I)	10–250 ppb	10 ppb
Optical sensor [39]	Fluorescent intensity	120 ppb–30 ppm	120 ppb
Electrochemical sensor [41]	Current (I)	0.5–120 ppb	0.5 ppb
Kovac’s or Ehrlich’s reagent [40]	Color	N/A	N/A

## Data Availability

Data are contained within the article.

## References

[1] Manges A. (2016). Escherichia coli and urinary tract infections: The role of poultry-meat. Clin. Microbiol. Infect..

[2] Addis M., Sisay D. (2015). A review on major food borne bacterial illnesses. J. Trop. Dis. Public Health.

[3] Tietjen M., Fung D.Y. (1995). Salmonellae and food safety. Crit. Rev. Microbiol..

[4] World Health Organization (2015). WHO Estimates of the Global Burden of Foodborne Diseases: Foodborne Disease Burden Epidemiology Reference Group 2007–2015.

[5] Yang L., Bashir R. (2008). Electrical/electrochemical impedance for rapid detection of foodborne pathogenic bacteria. Biotechnol. Adv..

[6] Mandal P., Biswas A., Choi K., Pal U. (2011). Methods for rapid detection of foodborne pathogens: An overview. Am. J. Food Technol..

[7] Swaminathan B., Feng P. (1994). Rapid detection of food-borne pathogenic bacteria. Annu. Rev. Microbiol..

[8] Law J.W.-F., Ab Mutalib N.-S., Chan K.-G., Lee L.-H. (2015). Rapid methods for the detection of foodborne bacterial pathogens: Principles, applications, advantages and limitations. Front. Microbiol..

[9] Gracias K.S., McKillip J.L. (2004). A review of conventional detection and enumeration methods for pathogenic bacteria in food. Can. J. Microbiol..

[10] Jenkins D.M., Kubota R., Dong J., Li Y., Higashiguchi D. (2011). Handheld device for real-time, quantitative, LAMP-based detection of Salmonella enterica using assimilating probes. Biosens. Bioelectron..

[11] You Y., Lim S., Hahn J., Choi Y.J., Gunasekaran S. (2018). Bifunctional linker-based immunosensing for rapid and visible detection of bacteria in real matrices. Biosens. Bioelectron..

[12] Lazcka O., Del Campo F.J., Munoz F.X. (2007). Pathogen detection: A perspective of traditional methods and biosensors. Biosens. Bioelectron..

[13] Sethi S., Nanda R., Chakraborty T. (2013). Clinical application of volatile organic compound analysis for detecting infectious diseases. Clin. Microbiol. Rev..

[14] Bayn A., Nol P., Tisch U., Rhyan J., Ellis C.K., Haick H. (2013). Detection of volatile organic compounds in brucella abortus-seropositive Bison. Anal. Chem..

[15] Lough F., Perry J.D., Stanforth S.P., Dean J.R. (2017). Detection of exogenous VOCs as a novel inávitro diagnostic technique for the detection of pathogenic bacteria. Trac Trends Anal. Chem..

[16] Bos L.D., Sterk P.J., Schultz M.J. (2013). Volatile metabolites of pathogens: A systematic review. Plos Pathog..

[17] Schnürer J., Olsson J., Börjesson T. (1999). Fungal volatiles as indicators of food and feeds spoilage. Fungal Genet. Biol..

[18] Ellis D.I., Goodacre R. (2001). Rapid and quantitative detection of the microbial spoilage of muscle foods: Current status and future trends. Trends Food Sci. Technol..

[19] Barth M., Hankinson T.R., Zhuang H., Breidt F. (2009). Microbiological Spoilage of Fruits and Vegetables. Compendium of the Microbiological Spoilage of Foods and Beverages.

[20] Tait E., Perry J.D., Stanforth S.P., Dean J.R. (2014). Bacteria detection based on the evolution of enzyme-generated volatile organic compounds: Determination of Listeria monocytogenes in milk samples. Anal. Chim. Acta.

[21] Senecal A.G., Magnone J., Yeomans W., Powers E.M. (2002). In Rapid Detection of Pathogenic Bacteria by Volatile Organic Compound (VOC) Analysis.

[22] Zhu J., Bean H.D., Kuo Y.-M., Hill J.E. (2010). Fast detection of volatile organic compounds from bacterial cultures by secondary electrospray ionization-mass spectrometry. J. Clin. Microbiol..

[23] Dolai S., Bhunia S.K., Beglaryan S.S., Kolusheva S., Zeiri L., Jelinek R. (2017). Colorimetric Polydiacetylene–Aerogel Detector for Volatile Organic Compounds (VOCs). ACS Appl. Mater. Interfaces.

[24] Eda G., Chhowalla M. (2010). Chemically derived graphene oxide: Towards large-area thin-film electronics and optoelectronics. Adv. Mater..

[25] Das Sarma S., Adam S., Hwang E.H., Rossi E. (2011). Electronic transport in two-dimensional graphene. Rev. Mod. Phys..

[26] Neto A.C., Guinea F., Peres N.M., Novoselov K.S., Geim A.K. (2009). The electronic properties of graphene. Rev. Mod. Phys..

[27] Warner J.H., Schäffel F., Bachmatiuk A., Rümmeli M.H. (2013). Graphene.

[28] Avouris P. (2010). Graphene: Electronic and photonic properties and devices. Nano Lett..

[29] Schwierz F. (2010). Graphene transistors. Nat Nanotechnol.

[30] Lin Z., Wu G., Zhao L., Lai K.W.C. (2019). Carbon nanomaterial-based biosensors: A review of design and applications. IEEE Nanotechnol. Mag..

[31] Huang Y., Dong X., Liu Y., Li L.-J., Chen P. (2011). Graphene-based biosensors for detection of bacteria and their metabolic activities. J. Mater. Chem..

[32] Mao H.Y., Lu Y.H., Lin J.D., Zhong S., Wee A.T.S., Chen W. (2013). Manipulating the electronic and chemical properties of graphene via molecular functionalization. Prog. Surf. Sci..

[33] Lin Y.-M., Dimitrakopoulos C., Jenkins K.A., Farmer D.B., Chiu H.-Y., Grill A., Avouris P. (2010). 100-GHz transistors from wafer-scale epitaxial graphene. Science.

[34] Meric I., Han M.Y., Young A.F., Ozyilmaz B., Kim P., Shepard K.L. (2008). Current saturation in zero-bandgap, top-gated graphene field-effect transistors. Nat. Nanotechnol..

[35] Dankerl M., Hauf M.V., Lippert A., Hess L.H., Birner S., Sharp I.D., Mahmood A., Mallet P., Veuillen J.Y., Stutzmann M. (2010). Graphene Solution-Gated Field-Effect Transistor Array for Sensing Applications. Adv. Funct. Mater..

[36] Ghoneim M.T., Smith C.E., Hussain M.M. (2013). Simplistic graphene transfer process and its impact on contact resistance. Appl. Phys. Lett..

[37] Wu J.B., Lin M.L., Cong X., Liu H.N., Tan P.H. (2018). Raman spectroscopy of graphene-based materials and its applications in related devices. Chem. Soc. Rev..

[38] Björk J., Hanke F., Palma C.-A., Samori P., Cecchini M., Persson M. (2010). Adsorption of aromatic and anti-aromatic systems on graphene through π− π stacking. J. Phys. Chem. Lett..

[39] Crunaire S., Marcoux P.R., Ngo K.-Q., Moy J.-P., Mallard F., Tran-Thi T.-H. (2014). Discriminating bacteria with optical sensors based on functionalized nanoporous xerogels. Chemosensors.

[40] Miller J.M., Wright J.W. (1982). Spot indole test: Evaluation of four reagents. J. Clin. Microbiol..

[41] Zhou Y., Ding M., Lyu W., Zhen Q., Chen H., Jiang M., Ding Y., Zhang X. (2021). A sensitive electrochemical method for indole based on the signal amplification strategy by gold/iron-oxide composite nanoparticles. Anal. Chim. Acta.

